# Pyroptosis in Respiratory Virus Infections: A Narrative Review of Mechanisms, Pathophysiology, and Potential Therapeutic Interventions

**DOI:** 10.3390/microorganisms13092109

**Published:** 2025-09-10

**Authors:** Runqi Lin, Barbara N. Porto

**Affiliations:** 1 Department of Medical Microbiology and Infectious Diseases, Rady Faculty of Health Sciences, University of Manitoba, Winnipeg, MB R3E 0J9, Canada; linr3@myumanitoba.ca; 2 Biology of Breathing Group, Children’s Hospital Research Institute of Manitoba, Winnipeg, MB R3E 3P4, Canada

**Keywords:** pyroptosis, cell death, respiratory virus, influenza virus, RSV, SARS-CoV-2, rhinovirus, human metapneumovirus, adenovirus

## Abstract

Pyroptosis is a mode of inflammatory cell death, characterized by cell membrane rupture and the release of pro-inflammatory cytokines and damage-associated molecular patterns (DAMPs). Pyroptosis is a critical part of the innate immune response and acts as a defense mechanism against different types of pathogens, including viruses. Several respiratory viruses, including influenza virus, respiratory syncytial virus (RSV), human metapneumovirus, and SARS-CoV-2, have been shown to trigger pyroptosis through distinct mechanisms. While pyroptosis is beneficial to the host by controlling virus replication and eliminating infected cells, the exaggerated induction of pyroptosis can be harmful and cause significant tissue damage, such as that to the lung tissue during infection with respiratory viruses. Therefore, understanding the mechanisms and the role pyroptosis plays during respiratory virus infections could lead to the development of novel therapeutic approaches to reduce the morbidity caused by these infections. In this review, we discuss the recent knowledge obtained on the pathophysiological role of pyroptosis during different respiratory viral infections as well as some experimental approaches to regulating its detrimental effects to the host.

## 1. Introduction

Pyroptosis is a lytic form of programmed cell death characterized by plasma membrane pore formation mediated by members of the gasdermin (GSDM) family of proteins [1]. In the canonical pathway of pyroptosis, intracellular pattern recognition receptors (PRRs), including the members of the Nod-like receptor family NLRP1, NLRP3, NLRC4, absent in melanoma 2 (AIM2), and pyrin, can form an inflammasome with pro-caspase-1 with the help of adaptor apoptosis-associated speck-like protein with CARD domain (ASC) [1]. The activation of the inflammasome leads to caspase-1 activation, which in turn induces the proteolytic cleavage of GSDMD, as well as the cleavage of pro-IL-1β and pro-IL-18 into active IL-1β and IL-18 [2,3,4]. GSDMD is cleaved into the N- and C-terminals, and the N-terminal then connects with phosphatidylinositol (PI) on the plasma membrane, resulting in oligomerization and pore formation [5]. This process results in cell swelling and lysis, with the consequent release of damage-associated molecular patterns (DAMPs) and pro-inflammatory cytokines including IL-1α, IL-1β, and IL-18 and ultimately pyroptosis [2,3,4,6,7,8]. Additionally, pyroptosis can be activated through several different pathways: in the non-canonical pathway, caspase-4/5 (caspase-11 in mice) instead of caspase-1 cleaves and activate GSDMD to induce pyroptosis, while in the caspase-3-dependent pathway, pyroptosis can be initiated through the caspase-3-mediated activation of GSDME [9,10,11]. Pyroptosis contributes to the induction of significant inflammatory responses and has been associated with various types of inflammatory, autoimmune, and infectious diseases [12,13,14,15,16].

**Figure 1:**
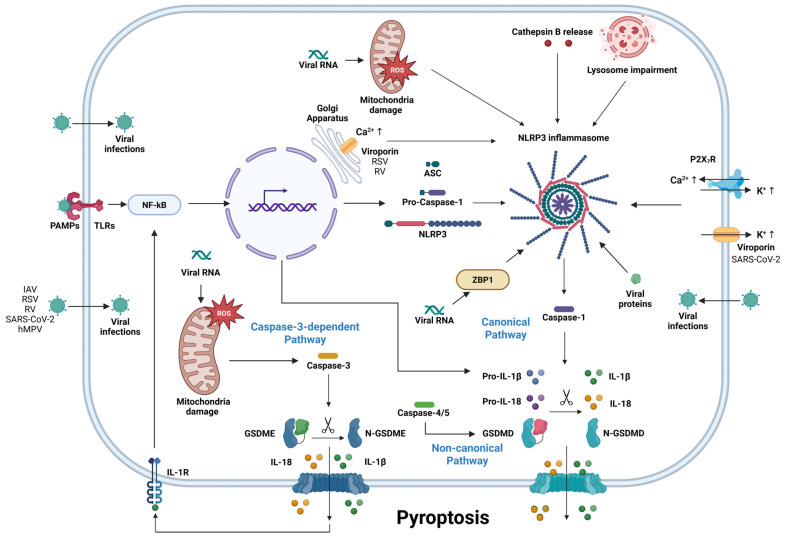
Pyroptosis activation by respiratory viruses. Recognition of viral infections by host receptors such as TLR leads to activation of NF-κB pathway, which results in upregulation of NLRP3, GSDMD, pro-IL-1β, and pro-IL-18. Multiple signals including viral-related cellular damage, viral genomes, and viral proteins can lead to assembly and activation of NLRP3 inflammasome. Following inflammasome activation, pro-caspase-1 is cleaved into its active form and facilitates cleavage of pro-IL-1β, pro-IL-18, and GSDMD to their active forms. Cleaved GSDMD (N-GSDMD) oligomerizes to form cell membrane pores, which allows for secretion of IL-1β and IL-18 and executes pyroptosis. Viruses can also execute pyroptosis through alternative pathways such as activation of GSDME by caspase-3. Created in BioRender. Lin, R. (2025) https://BioRender.com/9nzmq88.

The detection of pathogen-associated molecular patterns (PAMPs) and DAMPs during virus infection by host cell receptors can induce inflammasome activation and pyroptosis (Figure 1). The induction of pyroptosis is an important innate immune response that controls viral infection by removing virus replication niches, as well as inducing potent inflammatory responses [17]. Most respiratory viral infections typically cause mild cold-like symptoms; however, severe lower respiratory tract infections are often associated with bronchiolitis and viral pneumonia and can lead to fatal outcomes. Additionally, respiratory viruses could also play a role in asthma and chronic obstructive pulmonary disease (COPD) development and exacerbations, highlighting the detrimental role of hyper-inflammation in severe respiratory tract infections [18,19,20]. Indeed, several studies have suggested that respiratory virus infection can lead to severe inflammation and lung tissue damage through pyroptosis pathways [21,22,23].

In this review, we focus on respiratory viruses that have been responsible for outbreaks, epidemics, and pandemics, causing a significant impact on morbidity and mortality worldwide [24,25,26], including influenza virus, respiratory syncytial virus, SARS-CoV-2, and adenovirus, among others. We summarize the recent knowledge obtained on how these important respiratory viruses trigger pyroptosis, as well as the beneficial and detrimental roles of pyroptosis during respiratory viral infections. We also discuss some experimental approaches to reduce the morbidity caused by these infections.

## 2. Influenza A Virus

Influenza A virus (IAV) is a negative-sense, single-stranded RNA virus belonging to the *Orthomyxoviridae* family [27]. As the primary cause for influenza disease (flu) affecting up to 20% of the world population, severe infections are typically accompanied by increased lung inflammatory symptoms including bronchiolitis, alveolitis, and viral pneumonia [28,29]. Due to its high prevalence and morbidity, IAV is considered one of the most devastating viral infections in human history.

As pyroptosis is often triggered by inflammasome activation, the NLRP3 inflammasome’s role in disease pathogenesis has been well investigated in the context of IAV infection. Early studies infecting inflammasome-deficient (NLRP3-/-, ASC-/-, and CASP1-/-) mice with PR8 H1N1 virus have shown a significant reduction in survival rate, bronchoalveolar lavage (BAL) inflammatory cytokine secretion (IL-1β, IL-18, IL-6, TNF-α), and monocyte/neutrophil infiltration and increased IAV-associated lung necrosis [30,31]. A more recent study using NLRP3^R58w^ mice, which exhibit a hyperactivated NLRP3 inflammasome, also demonstrated an increased survival rate, lung inflammation (IL-1β, IL-17A), and viral clearance and decreased IAV-associated lung damage compared to WT mice [32]. The use of IL1r1-/- mice also provides evidence for lung neutrophil infiltration mediated by IL-1β as the main mechanism of protection against IAV mediated by the NLRP3 inflammasome [32]. However, NLRP3 inflammasome activation has also been associated with increased pathogenicity during IAV infection. For example, NRLP3- and caspase-1-deficient mice (NLRP3-/-, CASP1-/-) infected with highly pathogenic H7N9 IAV demonstrated a significantly increased survival rate, less weight loss, and overall reduced lung inflammation [33], suggesting that the NLRP3 inflammasome is detrimental to the host during H7N9 IAV infection. Interestingly, the effects of a temporal inhibition of the NLRP3 function during pathogenic IAV infection have shown that NLRP3 plays a dual role in infection [34]. While an early inflammatory response triggered by NLRP3 activation is protective against IAV, inflammasome activation in the later stages of infection contributes to disease pathogenesis and mortality [34]. NLRP3 inflammasome activation and the induction of pyroptosis, necroptosis, and apoptosis have been shown to be regulated by the interferon-inducible protein Z-DNA binding protein (ZBP1) in IAV-infected cells [35,36]. ZBP1 deficiency protected mice against IAV-induced mortality, lung inflammation, and airway epithelium damage [35,36]. Therefore, the induction of pyroptosis and, consequently, inflammatory injury during IAV infection seem to be controlled upstream of the NLRP3 inflammasome by ZBP1.

The role that the AIM2 inflammasome plays during IAV infection is conflicting. While Zhang et al. demonstrated that AIM2-/- mice infected with both PR8 and CA07 IAV exhibited reduced lung injury and inflammation, as well as an overall increased survival rate compared to IAV-infected WT mice [37], Schattgen et al. reported that PR8 IAV infection significantly reduced the survival rate of AIM2-/- mice compared with WT mice [38]. This discrepancy could be attributed to differences in experimental conditions between the two studies, such as the cell culture systems used for viral propagation and virus dosage [37,38].

GSDMD is a crucial component of inflammasomes and a key effector of pyroptosis [39]. Two recent studies have investigated the role of GSDMD in IAV-induced disease pathogenesis. These studies demonstrated that a lack of GSDMD is associated with a significantly improved survival rate and reduced disease severity during IAV infection. Also, IAV-induced lung tissue damage and inflammation are impaired in GSDMD-deficient mice, particularly in neutrophil infiltration [22,40]. These studies suggest that GSDMD plays a major role in promoting IAV disease severity through neutrophil-associated inflammation. IAV is also able to induce the pyroptosis of lung alveolar epithelial cells through the activation of the caspase-3/GSDME pathway [41,42]. GSDME-/- mice were shown to be protected against lethal H7N9 infection with improved survival rates and reduced lung immunopathology and inflammation [41]. Baicalin, a compound found in an ingredient of Traditional Chinese Medicine, was shown to improve survival and reduce the lung immunopathology of mice infected with H1N1 through the inhibition of the caspase-3/GSDME pathway [42]. Collectively, these studies point to the detrimental role of GSDMD- and GSDME-mediated pyroptosis in IAV infection and suggest that targeting GSDMD and/or GSDME may provide a novel therapeutic option for treating severe influenza infection.

Due to the frequent occurrence of secondary bacterial infections during IAV infection, several studies also investigated the role of pyroptosis during IAV-associated secondary bacterial infections. Robinson et al. investigated the role of the inflammasome in IAV and *Staphylococcus aureus*-co-infected mice. They observed that the co-infection of ASC-/- mice resulted in reduced lung neutrophil and macrophage recruitment as well as reduced inflammation, while more effective bacterial clearance was observed compared to WT mice [43]. Interestingly, it was also reported that the inhibition of the NLRP3 inflammasome with NLRP3 inhibitor MCC950 only in the late stages of the infection reduced bacterial burden, while early inhibition increased burden, further highlighting the time-dependent role of inflammasome activation in IAV infection outcomes [43]. More recently, You et al. investigated the role of pyroptosis during IAV and *Streptococcus pneumoniae* co-infections. Infection with both IAV and *S. pneumoniae* suppressed the expression of the E3 ubiquitin ligase NEDD4 in human airway epithelial cells. Immunoprecipitation and siRNA inhibition of NEDD4 revealed that NEDD4 directly interacts with and differentially regulates the expression of NLRP3 and GSDMD. While the inhibition of NEDD4 resulted in increased NLRP3 expression, NEDD4 inhibition led to a decreased expression level of GSDMD [44]. Overall, this study suggests that NEDD4 may play an intricate and important role in regulating pyroptosis initiation and activation during IAV and *S. pneumoniae* co-infection, and further studies are required to understand the mechanisms of this regulation.

A growing body of evidence has shown the crosstalk between different modes of cell death [45,46,47], but the exact molecular mechanisms that determine the concomitant activation of proteins involved in multiple cell death pathways are not well understood. IAV has been reported to promote apoptosis and pyroptosis but not necroptosis in human bronchial epithelial cells. While apoptosis is triggered in the early stages of infection, pyroptosis is induced in later stages [48]. Interestingly, type-I interferon (IFN)-mediated JAK-STAT signaling promotes the switch from apoptosis to pyroptosis in respiratory epithelial cells to initiate an inflammatory response to IAV infection [48]. However, whether this inflammation is protective or harmful to the host during IAV infection was not addressed in this study. In 2020, a cell death complex composed of key molecules from pyroptosis, apoptosis, and necroptosis, called the PANoptosome, was identified in mouse bone marrow-derived macrophages (BMDMs) after infection with IAV and shown to be responsible for triggering PANoptosis, an inflammatory cell death that occurs through the collective activation of pyroptosis, apoptosis, and necroptosis [49]. The discovery of the PANoptosome shifted the traditional view on cell death pathways being mutually exclusive. The crosstalk between different cell death modes could provide advantages against pathogen evasion mechanisms from the host immune response. On the other hand, the simultaneous activation of multiple inflammatory cell death pathways, such as necroptosis and pyroptosis, could further exaggerate pathogen-associated excessive tissue inflammation.

Overall, IAV infection-induced pyroptosis has been shown to have both beneficial and detrimental roles and can either protect the host against IAV or worsen disease outcomes. While most of the studies suggest that pyroptosis plays a detrimental role during IAV infection, the specific role of pyroptosis is likely dependent on many factors including IAV strains, viral load, and the stage of the infection. Therefore, all these factors should be taken into consideration when targeting pyroptosis for IAV infection treatment.

## 3. Respiratory Syncytial Virus

Respiratory syncytial virus (RSV) is a negative-sense, single-stranded RNA virus belonging to the *Pneumoviridae* family [50]. RSV is one of the most common viral infections in infants and young children [51,52]. While RSV infection can cause mild and cold-like symptoms, babies, young children, and older adults have a higher risk of developing severe symptoms such as bronchiolitis and viral pneumonia [53]. RSV accounts for over 2% of all deaths of children under 5 years old annually [24]. Additionally, severe RSV bronchiolitis in childhood is associated with an increased risk of developing asthma and allergies in adulthood [54]. It is undeniable that RSV poses a significant burden to global pediatric health.

Early studies have demonstrated that RSV infection of human neonatal monocytes and airway epithelial cells results in increased expression levels of caspase-1 and IL-1β and suggested the potential of targeting this pathway for the treatment of respiratory tract infections [55,56]. Segovia et al. investigated the mechanisms of RSV-induced IL-1β secretion. The authors reported that the TLR2/MyD88/NF-κB pathway, reactive oxygen species (ROS), and K^+^ efflux are required for NLRP3/ASC inflammasome activation and IL-1β processing/secretion, following RSV infection in human and mouse macrophages [57]. However, in primary human lung epithelial cells, TLR4 is required for IL-1β maturation, and the activation of the NLRP3 inflammasome is induced by RSV small hydrophobic (SH) viroporin [58]. These studies suggest that the NLRP3 inflammasome activation mechanisms induced by RSV infection differ depending on the cell type. The role of IL-1β in RSV pathology was also investigated. IL-1β has been reported to induce the production of inflammatory cytokines such as thymic stomal lymphopoietin (TSLP), CCL2, and IL-33 following RSV infection, and these cytokines can contribute to RSV-induced immunopathology [59,60]. Importantly, the inhibition of IL-1β signaling with an IL-1 receptor antagonist (IL1-RA) in RSV-infected neonatal mice reduces RSV-mediated lung immunopathology and inflammation, as well as reducing CCL5, IL-4, and neutrophil infiltration [61]. Interestingly, treatment with IL1-RA also greatly alleviates subsequent asthma development using the cockroach antigen (CRA) allergy challenge model [61]. The role of the NLRP3 inflammasome in RSV pathogenesis was also directly investigated. Malinczak et al. demonstrated that NLRP3-deficient mice and mice treated with the NLRP3 inhibitor MCC950 both exhibited decreased RSV-mediated lung immunopathology and inflammation, with decreased IL-1β, IL-33, CCL2, and IL-13 secretion [23]. Interestingly, neonate mice infected with RSV and treated with MCC950 displayed reduced RSV-exacerbated allergic responses [23]. Recently, Jiao et al. demonstrated that berberine, a compound previously shown to reduce RSV-mediated inflammation and replication in human airway epithelial cells [62,63], can inhibit NLRP3 inflammasome-mediated pyroptosis both in RSV-infected human airway epithelial cells and RSV-infected mice, which is accompanied by reduced IL-1β and IL-18 release and lung injury [64].

Together, these studies support the idea that the NLRP3 inflammasome-induced pyroptosis pathway can contribute to the increased lung immunopathology induced by RSV infection as well as RSV-associated asthma development. However, many aspects of pyroptosis still warrant investigation, such as the role of the non-canonical pyroptosis pathway, as well as the role of pyroptosis at different stages of RSV infection.

## 4. Rhinovirus

Rhinovirus (RV) is a positive-sense, single-stranded RNA virus belonging to the *Picornaviridae* family [65]. Rhinovirus is the most frequent cause of upper respiratory tract infections and common cold, but it can also cause lower respiratory tract infections and lead to severe symptoms such as bronchiolitis and viral pneumonia [66,67]. Currently, rhinovirus is categorized into three species, RV-A, RV-B, and the most recently discovered RV-C, which has been associated with more severe illness [66,68]. Importantly, rhinovirus infection can also lead to virus-induced asthma exacerbations, and early-life rhinovirus-induced wheezing has been associated with asthma development [69].

Several studies have investigated the role of pyroptosis pathways in RV infections and RV-associated asthma. Different types of inflammasomes have been implicated in RV-induced pyroptosis in human airway epithelial cells. Early studies have identified rhinovirus viroporin 2B as an inducer of calcium ion imbalance, which triggers the activation of NLRP3 and NLRC5 inflammasomes and subsequent IL-1β release [70]. NLRP3 inflammasome-mediated pyroptosis was later shown to be activated by rhinovirus through the DDX_33_/RIG-I–NLRP3–caspase-1–GSDMD axis [71], while RIG-I-mediated inflammasome activation independent of NLRP3 was also reported in primary human airway epithelial cells [72]. More recently, Laanesoo et al. also identified prominent NLRP1 inflammasome activation and IL-1β/IL-18 secretion upon rhinovirus infection in primary human airway epithelial cells [73].

The roles of pyroptosis-induced IL-1β and IL-18 in RV infection have been well investigated. RV infection-induced IL-1β was reported to increase the downstream expression of IL-6, colony-stimulating factor 2 (CSF2), CXCL2, and CXCL8 in human airway epithelial cells, which suggests the potential of IL-1β signaling in inducing severe disease during RV infection [74,75]. In contrast, experimental RV infection in healthy and asthmatic subjects showed that increased baseline mucosal levels of IL-18 but not IL-1β are correlated with decreased respiratory symptoms, suggesting that early pyroptosis activation and the expression of mucosal IL-18 could potentially provide a protective effect against RV infection [76]. The difference observed in the role of Il-1β and IL-18 in RV infection could be attributed to several factors. For example, IL-18, also known as interferon-gamma-inducing factor, is known to promote IFN-γ production in different cells, which can exert direct antiviral responses [77]. IL-1β, but not IL-18, induces the expression of cyclooxygenase 2 (COX-2), which is responsible for the production of prostaglandins that play a prominent role in mediating acute tissue inflammation [78,79]. Moreover, IL-18 signaling also utilizes the IL-18 receptor, which is distinct from the IL-1 receptor [80]. Finally, the production of IL-18, but not IL-1β, is also dependent on type-I interferon signaling [81], which could partially contribute to the outcomes observed from the experimental RV infection in humans, as higher baseline IL-18 levels could be associated with higher levels of type-I interferons.

The roles of inflammasome activation in RV infection have also been directly studied. In human nasal epithelial cells (HNECs), RV-induced NLRP3 inflammasome activation has been reported to increase the production of epithelial mucin protein MUC5AC [71]. Moreover, nasal tissue from chronic rhinosinusitis (CRS) patients with RV infection exhibits increased NLRP3 and IL-1β expression compared to CRS patients without RV infection [71], suggesting that NLRP3 inflammasome-mediated pyroptosis could contribute to mucus hypersecretion and airway chronic inflammation during RV infection. In the same study, the authors also reported that the inhibition of NLRP3 inflammation activation prior to RV infection drastically increased RV replication, indicating that early inflammasome activation can play an important role in suppressing RV replication in human airway epithelial cells [71]. However, Radzikowska et al. later demonstrated that RV-induced RIG-I inflammasome activation is associated with delayed RV clearance both in asthmatic patients and in in vitro experiments. They hypothesized that RIG-I inflammasome activation by rhinovirus could delay viral clearance in patients by interfering with RIG-I-dependent interferon signaling pathways [72]. Finally, the NLRP3 inflammasome and IL-1β signaling in RV-associated asthma were extensively investigated. NLRP3 and IL-1β inhibition in mice infected with RV resulted in an asthma-like phenotype, including increased mucus production and type 2 inflammation in immature mice compared to mature mice [82,83]. In contrast, NLRP3 and IL-1β inhibition in mature mice led to a reduction in the asthma-like phenotype [82,83], suggesting that the NLRP3 inflammasome and its subsequent IL-1β signaling could either be beneficial or detrimental in RV-associated asthma development and exacerbation depending on the subject’s developmental status. Furthermore, Han et al. compared the effects of RV-A and RV-C infection in immature mice and found that inflammasome activation and IL-1β release were reduced in RV-C infection compared with RV-A infection, while RV-C-infected mice developed a more severe asthmatic phenotype [84]. Additionally, exogenous IL-1β treatment reduced the RV-C-mediated asthma-like phenotype in immature mice [84].

Overall, pyroptosis and its associated IL-1β signaling have been shown to influence rhinovirus disease severity both beneficially and detrimentally. However, further investigation is required to determine the underlying roles of pyroptosis activation at different stages of RV infection. Additionally, inflammasome activation and IL-1β signaling can contribute to the development of rhinovirus-induced asthma, depending on patients’ developmental status. This could provide insights into developing personalized therapies against RV-associated asthma development and exacerbations.

## 5. SARS-CoV-2

First discovered in 2019, in Wuhan, China, SARS-CoV-2 is a positive-sense, single-stranded RNA virus that belongs to the *Coronaviridae* family [85]. A combination of factors, including SARS-CoV-2 being a newly emerged pathogen without prior immunity in humans, its aerosol mode of transmission, and efficiency in transmission, all led to the global spread of the virus and the coronavirus disease 2019 (COVID-19) global pandemic [86]. SARS-CoV-2 infection has a varied spectrum of clinical presentations, ranging from asymptomatic infections to acute respiratory failure, sepsis, and multiorgan failure [87]. A key clinical feature often observed in severe COVID-19 patients is a dysregulated immune response with innate immune hyperactivation and underwhelming secondary immune response along with symptoms including cytokine storm, neutrophilia, and lymphopenia [88]. Therefore, investigating the role of innate immune responses during SARS-CoV-2 infection has been a major area of research aiming to better understand COVID-19 pathogenesis.

Early clinical studies observed signs of inflammasome activation (caspase-1 activation, IL-1β and IL-18 secretion), as well as increased LDH release (a sign of lytic cell death) in the sera of severe COVID-19 patients, suggesting the potential involvement of pyroptosis in severe SARS-CoV-2 infection [89,90,91,92,93]. Numerous studies have since detailed the underlying mechanisms of inflammasome and pyroptosis activation and inhibition by SARS-CoV-2, and NLRP3 has been shown to be the primary sensor involved [94,95]. The SARS-CoV-2 spike (S) protein, known for its role in binding with the cell surface receptor ACE2, has been shown to activate the NLRP3 inflammasome and trigger IL-1β release from both macrophages and microglia derived from COVID-19 patients and healthy donors through TLR2-mediated signaling [94,95]. On a similar note, the SARS-CoV-2 S protein can also mediate the syncytium formation of ACE2-expressing human airway epithelial cells and the subsequent pyroptosis of the syncytia through the caspase-3/GSDME-mediated pathway [96]. Additionally, SARS-CoV-2 non-structural protein 6 (NSP6) activates NLRP3 inflammasome-mediated pyroptosis along with IL-1β and IL-18 maturation and secretion in human airway epithelial cells. Mechanistically, NSP6 can directly inhibit a subunit of the vacuolar ATPase protein pump ATP6AP1, resulting in the impairment of lysosome acidification and autophagic flux, which leads to pyroptosis [97,98]. Ambrozek-Latecka et al. demonstrated that SARS-CoV-2 viroporin ORF3a, envelope (E), and membrane (M) proteins can also induce NLRP3 inflammasome activation and pyroptosis in human macrophages [99]. While ORF3a has been previously shown to activate the NLRP3 inflammasome through K^+^ ion efflux [100], the mechanisms by which E and M proteins trigger pyroptosis require further investigation. Interestingly, the finding of E protein-mediated NLRP3 activation contrasts with a prior study by Yalcinkaya et al., in which they reported that the SARS-CoV-2 E protein suppressed NLRP3 inflammasome activation in human macrophages and mice [101]. Several other SARS-CoV-2 proteins have been shown to regulate host NLRP3 inflammasome-mediated pyroptosis. SARS-CoV-2 NSP1 and NSP13 were reported to potently inhibit NLRP3-mediated caspase-1 activity and IL-1β secretion [102]. The SARS-CoV-2 nucleocapsid (N) protein’s role in pyroptosis is conflicting. While Pan et al. reported that the N protein directly binds to NLRP3, promoting NLRP3 inflammasome activation in airway epithelial cells and monocyte-derived macrophages [103], Ma et al., on the other hand, demonstrated that the N protein modulates pyroptosis by binding to GSDMD and the binding of the N protein at the linker region of GSDMD near D^265^ results in the blockage of the interaction between GSDMD and caspase-1, which prevents its activation by caspase-1 [104], suggesting that the N protein can play an intricate role in pyroptosis regulation. Finally, the NLRP1 inflammasome is also involved in SARS-CoV-2-mediated pyroptosis. The viral protease NSP5 has been shown to both activate NLRP1 inflammasome assembly and inactivate GSDMD in lung epithelial cells through the proteolytic cleavage of GSDMD at site Q^193^, resulting in the production of inactive GSDMD fragments, thereby pushing the cells into pyroptosis through the NLRP1 inflammasome-mediated caspase-3/GSDME pathway [105].

Zeng et al. reported that the inhibition of the NLRP3 inflammasome in SARS-CoV-2-infected mice using the NLRP3 inhibitor MCC950 resulted in decreased signs of interstitial pneumonia, reduced expression of pro-inflammatory cytokines and chemokines (IL-1β, IL-6, IL-18, TNA-α, CCL5, and CXCL8), and overall reduced lung immune cell infiltration [21]. In addition, an early meta-analysis of four non-randomized cohort studies revealed that the use of the IL-1 receptor antagonist anakinra in patients with COVID-19 is also associated with significantly reduced mortality (10% vs. 41% in the control group) and the need for mechanical ventilation (15% vs. 36% in the control group) [106]. Furthermore, a phase 3 clinical trial conducted using anakinra in COVID-19 patients with a high risk of respiratory failure development revealed that the administration of anakinra resulted in overall improved clinical outcomes and decreased mortality compared with placebo treatment [107]. These studies suggest an overall detrimental role of pyroptosis and its subsequent IL-1β signaling in SARS-CoV-2 infection. During SARS-CoV-2 infection, lung inflammasome activation and pyroptosis potently release IL-1β, IL-6, IL-18, and CXCL8, which induces neutrophil recruitment to lung tissue and plays an important role in mediating lung immunopathology [108].

Furthermore, pyroptosis also induces neutrophil-mediated lung immunopathology. Silva et al. discovered that the formation of SARS-CoV-2-mediated neutrophil extracellular traps (NETs) is dependent on GSDMD activation, and treatment with the GSDMD inhibitor disulfiram inhibited the human airway epithelial and endothelial cell damage induced by neutrophils infected with SARS-CoV-2. GSDMD also mediated SARS-CoV-2-induced lung immunopathology in mice, further supporting the idea that pyroptosis may worsen COVID-19 disease by promoting neutrophil-mediated lung immunopathology [109]. This evidence also suggests that pyroptosis may be one of the drivers of COVID-19-associated immunothrombosis, as NET release and pyroptosis-associated endothelial cell death and its subsequent signaling have been linked with thrombosis in COVID-19 patients [110,111]. Moreover, the deletion of caspase-11, a mice homolog of the human non-canonical inflammasome caspase-4, has been shown to be protective against SARS-CoV-2-induced lung coagulopathy in mice [112]. Outside of the respiratory tract, Junqueira et al. demonstrated that SARS-CoV-2 can infect circulating monocytes and induce pyroptosis and inflammatory cytokine secretion via the NLRP3/AIM2–caspase-1–GSDMD pathway, highlighting a mechanism by which SARS-CoV-2-mediated pyroptosis can induce systemic inflammation and cytokine storm through circulating monocytes [113]. Pyroptosis has previously been shown to contribute to various organ-specific pathologies, such as nonalcoholic fatty liver disease, myocardial ischemia, and acute kidney injuries [114,115,116]. However, despite accumulating evidence for inflammasome activation at various tissues such as endothelial cells and cardiomyocytes [99,117], there is currently a lack of evidence to support GSDM activation-mediated cell death as a result of SARS-CoV-2 infection in non-pulmonary organ tissues outside of the spleen [118].

However, a recent report by Bader et al. provided evidence against the role of pyroptosis in mediating SARS-CoV-2 pathogenesis. In this study, the authors show that NLRP3-/-, NLRP1-/-, ASC-/-, GSDMD-/-, GSDME-/-, GSDMA/C/D/E-/-, and caspase-1/11/12-/- mice exhibited no significant differences in viral load and disease severity compared with infected WT mice. While only ASC-/- and GSDMD-/- mice exhibited a slight reduction in lung IL-1β expression during SARS-CoV-2 infection, the infection of IL-1β-/- mice resulted in reduced lung viral burden, pathology, and weight loss [119]. These results are in clear contrast with the previous study in mice targeting the NLRP3 inflammasome by Zeng et al. [21]. Different models of SARS-CoV-2 infection could contribute to the discrepancies between the studies: Zeng et al. pre-infected mice with adenovirus-associated virus containing *hACE2*, which allows for SARS-CoV-2 infection in C57BL/6J mice [21], while Bader et al. infected C57BL/6J mice with a mouse-adapted SARS-CoV-2 containing spike N501Y mutation, which allows for viral infection using murine ACE2 [119,120]. The introduction of adenoviral vectors and the adaptation of SARS-CoV-2 to mice could lead to different host immune responses against SARS-CoV-2 infection. Overall, this study challenged the role of pyroptosis and further reinforced the importance of IL-1β signaling in COVID-19 disease pathogenesis while highlighting the need to better understand inflammasome-independent IL-1β processing.

Evidence so far supports that pyroptosis and subsequent IL-1β signaling play an important role in mediating COVID-19 disease pathology and can contribute to COVID-19 disease severity through multiple mechanisms, including lung immunopathology, systemic inflammation, and thrombosis. While recent evidence challenged the role of inflammasome and pyroptosis activation in mediating SARS-CoV-2 disease severity, it further highlights the need for a better understanding of lytic cell death mechanisms and their role in antiviral immune responses.

## 6. Human Metapneumovirus

Similarly to respiratory syncytial virus, human metapneumovirus (hMPV) is a single-stranded, negative-sense RNA virus that belongs to the *Pneumoviridae* family and is known to cause respiratory infection in infants, older adults, and immunocompromised individuals [121,122]. hMPV infection typically presents with common cold-like symptoms, while severe hMPV infection can lead to the development of more detrimental symptoms including bronchiolitis and pneumonia [121,122].

The roles of the inflammasome and pyroptosis in hMPV infection were first investigated by Malmo et al. In this study, the authors found a significantly increased expression level of the pyroptosis-associated cytokine IL-18 in nasopharynx aspirates from hMPV-infected children, as well as large variation in IL-1β, NLRP3, and IκBα expression among hMPV-infected patients [123]. A more frequent upregulation of IL-1β and NLRP3 was seen in patients with more severe hMPV infection with severity scores of 1 to 4 compared to patients with severity scores of 0 [123]. This study suggests that while hMPV-associated pyroptosis may depend on various host factors, it could also be associated with more severe hMPV infections [124]. The direct role of NLRP3 was later investigated by Le et al., in which the authors reported that treatment with MCC950 protected mice from hMPV clinical isolate C85473-induced mortality and weight loss [124]. The treatment also reduced hMPV-induced IL-1β, IL-6, IFN-γ, and TNF-α production without impacting viral titer [124]. Importantly, they found that mice lacking IL-1β, as well as mice infected with an hMPV strain missing the small hydrophobic (SH) protein, whose infection led to reduced caspase-1 activation in vitro, had less severe disease compared to WT mice [124]. This suggests that hMPV can trigger NLRP3 inflammasome-mediated pyroptosis through its SH protein and that this pathway may worsen disease by promoting IL-1β-driven inflammation independent of hMPV replication. However, a recent study by Zhang et al. found a different outcome. The authors observed no differences in disease severity in ASC- and NLRP3-deficient mice infected with different hMPV clinical isolates (TN94/49, C2-202, TN94-344, TN/91-320) compared to WT mice infected with the same isolates [125]. These differences could result from a combination of factors including distinct viral isolates used between the studies, as well as differences in the strains mice used: Le et al. infected BALB/c with hMPV, which has been associated with more severe clinical outcomes following hMPV infection compared to the C57BL/6 mice used by Zhang et al. [124,126]. Lastly, IL-1β has been shown to promote hMPV replication through the cGAS-STING pathway [127]. It was suggested by the authors that the IL-1β-cGAS-STING pathway could promote early hMPV replication through an unknown mechanism to avoid early type-I IFN production and subsequent antiviral signaling [127].

Investigating the role of IL-1β in hMPV-associated bacteria co-infection has yielded interesting results. Loevenich et al. discovered that while bacteria stimuli themselves (LPS, *E. coli*, *P. auruginosa*, *S. pneumoniae*) triggered a drastic increase in the expression of IL-1β in human macrophages, pre-infection with hMPV significantly decreased bacteria stimuli-induced IL-1β expression [128]. This inhibition of IL-1β expression was found to be attributed to secreted IFN-β following hMPV infection, where secreted IFN-β epigenetically repressed the *IL1B* promoter, resulting in decreased IL-1β transcription, which could contribute to increased susceptibility to secondary bacterial infections [128]. Overall, this study uncovered important insights into how respiratory viruses can modulate host inflammasome-associated inflammatory response, thereby increasing susceptibility for secondary infections.

While various factors, including host factors and different viral strains, can contribute to contrasting infection outcomes, evidence so far suggests that pyroptosis can lead to worse hMPV disease outcomes through IL-1β-mediated inflammation and increased hMPV replication in airway epithelial cells. Further investigation is warranted to better characterize the pyroptosis pathway during hMPV infection and study potential therapeutic targets against hMPV.

## 7. Adenovirus

Adenoviruses are family of double-stranded DNA viruses with currently over 50 different serotypes that can infect humans [129]. While human adenoviruses (HAdVs) can cause a variety of clinical manifestations, they frequently cause respiratory tract infections that typically lead to cold-like symptoms in both children and adults [130]. Additionally, engineered adenoviruses are commonly used as delivery vectors for gene therapy, vaccination, and anticancer agents [131]. Therefore, numerous studies have focused on the interactions between adenovirus and host innate immune responses.

Previous studies have shown that human adenovirus 5 (HAdV-5) induces NLRP3-mediated, cathepsin-B-dependent pyroptosis in human macrophages potentially through the TLR-9 sensing of viral dsDNA [132,133]. Labzin et al. demonstrated that antibody-opsonized HAdV-5 can induce inflammasome activation through different pathways. For example, the phagocytosis of antibody-bound HAdV-5 can result in lysosomal damage and NLRP3 inflammasome activation [134]. Antibody-opsonized HAdV-5 can also be detected by TRIM21, a cytosolic antibody receptor, which mediates viral capsid degradation, and the detection of the viral genome in cytosol can also trigger NLRP3 inflammasome activation [134]. In dendritic cells, HAdV-5 and neutralizing antibody immune complexes have also been reported to induce AIM2 inflammasome-mediated pyroptosis [135]. Moreover, HAdV complexes with the host antimicrobial proteins lactoferrin and α-defensin-1, leading to TLR-4-mediated internalization and NLRP3-mediated IL-1β release without lytic cell death [136,137]. The 100 kDa protein encoded by the L4 gene of HAdV-7 can also directly interact and activate NLRP3 inflammasome for IL-1β release [138]. In contrast, the viral-associated RNAI of HAdV-5 has been reported to directly inhibit NLRP3 inflammasome assembly in human macrophages by blocking the function of protein kinase R (PKR) [139], which has been implicated in mediating NLRP3 inflammasome assembly [140]. Finally, the murine AdV3 infection of murine-derived macrophages revealed the suppression of ASC-dependent inflammasome activation, thereby dampening host innate immune response and potentially increasing susceptibilities to secondary bacterial infections [141].

A recent clinical study has observed that HAdV-infected patients with ARDS exhibited a significantly increased expression level of IL-1α and IL-1β. In particular, an increased expression level of IL-1β was associated with increased disease severity and worse lung function, which suggested a potential value for IL-1β in predicting HAdV disease severity and progression [142]. In vitro experiments by Li et al. demonstrated that the HAdV-3 infection of human macrophages can lead to caspase-4 and caspase-5 non-canonical inflammasome-mediated pyroptosis and IL-1β release dependent on NF-κB [143]. The inhibition of caspase-4, caspase-5, and GSDMD greatly reduced the extracellular HAdV-3 titer in vitro, suggesting that caspase-4- and caspase-5-mediated pyroptosis could play a role in promoting HAdV-3 viral release [143]. In contrast, caspase-1-deficient and ATP receptor P2X_7_R-deficient mice infected with HAdV display improved survival compared with WT mice, which correlates with decreased IL-1β and IL-6 secretion and neutrophil infiltration [144]. This study suggested the role of the ATP receptor in HAdV-induced pyroptosis but also the potential detrimental role of pyroptosis and its downstream inflammatory signaling in promoting severe HAdV lung infection [144].

Overall, different serotypes of adenovirus have been shown to activate pyroptosis through a variety of mechanisms in macrophages and dendritic cells. While innate immune signaling mediated by adenovirus-induced pyroptosis in antigen-presenting cells would likely help initiate effective adaptive immune responses following adenovirus infection, evidence suggests that in the context of respiratory adenovirus infection, pyroptosis could promote virus spread by inducing virion release. Additionally, downstream innate immune signaling could lead to lung immunopathology associated with respiratory adenoviral infections.

## 8. Conclusions

**Figure 2:**
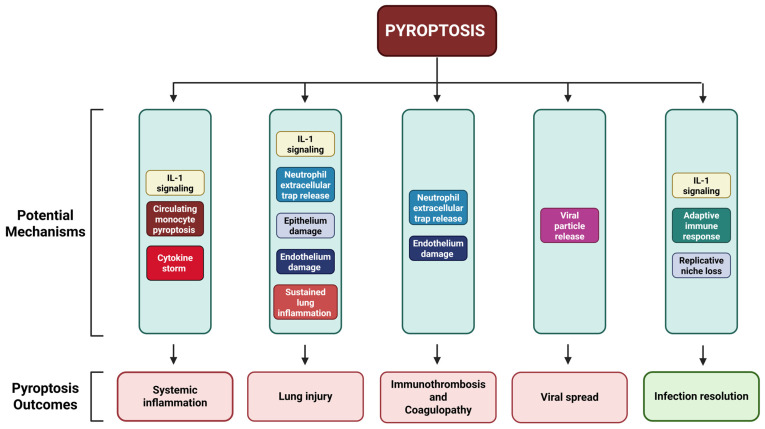
Roles of pyroptosis in respiratory viral infection outcomes. Pyroptosis activation during respiratory viral infections can lead to several outcomes. Pyroptosis activation can lead to effective elimination of viral replicative niche, induce effective innate immune responses, and promote adaptive immune responses to resolve infections. However, uncontrolled pyroptosis can result in various detrimental outcomes such as promoting virus release, immunothrombosis, lung immunopathology, and systemic inflammation. Created in BioRender. Lin, R. (2025) https://BioRender.com/2mwago3.

Several factors such as different viral strains, viral load, and age of patients can influence the roles played by pyroptosis during respiratory viral infections. Notably, numerous studies have suggested that pyroptosis may play distinct roles depending on the relative timing of viral infection. Pyroptosis activation during initial and early respiratory viral infections could limit virus replication and promote virus clearance, inducing potent inflammatory responses and promoting antigen presentation, which results in adaptive immune responses to resolve infection. This could explain the numerous adaptations in respiratory viruses to inhibit pyroptosis activation. However, the uncontrolled activation of pyroptosis at later stages of viral infections and consequently exaggerated inflammation can be detrimental to the host, causing lung tissue damage, which could lead to cytokine storm, bronchiolitis, and pneumonia (Figure 2). Therefore, targeting pyroptosis is an attractive therapeutic option against several common respiratory viruses. Nevertheless, a carefully timed and targeted blockade of pyroptosis and its associated pathways is critical to avoid unwarranted effects to the host. Moreover, a further understanding of the effects of pyroptosis at different stages of viral infections may prove beneficial for clinicians to develop case-specific treatments for patients by selectively targeting pyroptosis pathways for more effective treatment.

Additionally, mouse models have been heavily utilized to investigate the role of pyroptosis in respiratory viral infections and provided important mechanistic insights into inflammasome biology and pyroptosis pathophysiology. Multiple studies have highlighted the differences between mouse and human inflammasomes, particularly in their divergence in inflammasome sensors and tissue-specific responses against stimuli [145,146,147,148]. Given the differences on inflammasome and pyroptosis signaling between mice and humans, caution is needed when translating research into therapeutic development for human disease. Current treatments for viral infections are limited and can have broad effects on the host. Disulfiram remains the only FDA-approved drug that directly inhibit pyroptosis activation. Mechanistically, disulfiram covalently modifies Cysteine 191 in GSDMD, which prevents its ability to oligomerize and form lytic pores on cell membranes, thereby preventing GSDMD-mediated pyroptosis [149]. Thus, disulfiram could be repurposed to treat respiratory viral infections and other inflammatory diseases. However, disulfiram has also been suggested to possess inhibitory properties in multiple inflammatory pathways, including TLR4, NF-κB, and the NLRP3 inflammasome [150,151,152], which could potentially influence off-target pathways that could aid in pathogen clearance. Other therapeutic options, such as IL-1 signaling inhibitor anakinra, have shown no difference in effectiveness compared with the standard of care in severe COVID-19 patients with pneumonia [153]. Overall, pyroptosis and its associated cytokine signaling can play an important role in determining respiratory viral disease outcomes. Therefore, understanding the characteristics and influences of different pyroptosis pathways during respiratory viral infections and targeting pyroptosis pathways and downstream cytokine signaling could lead to the development of novel therapeutic approaches against common and emerging respiratory pathogens.

## Data Availability

No new data were created or analyzed in this study.
